# A Novel Coincidence: Essential Thrombocythemia with Facioscapulohumeral Muscular Dystrophy

**DOI:** 10.4274/tjh.galenos.2020.2020.0363

**Published:** 2020-11-19

**Authors:** Ceren Hangül, Orhan Kemal Yücel, Aslı Toylu, Hilmi Uysal, Sibel Berker Karaüzüm

**Affiliations:** 1 Akdeniz University Faculty of Medicine, Department of Medical Biology and Genetics, Antalya, Turkey; 2 Akdeniz University Faculty of Medicine, Department of Hematology, Antalya, Turkey; 3 Akdeniz University Faculty of Medicine, Department of Medical Genetics, Antalya, Turkey; 4 Akdeniz University Faculty of Medicine, Department of Neurology, Antalya, Turkey

**Keywords:** Essential thrombocythemia, ET, Facioscapulohumeral Muscular Dystrophy, FSHD, JAK2 p.V617F mutation

## To the Editor,

Essential thrombocythemia (ET) is a myeloproliferative disorder with elevated numbers of thrombocytes and facioscapulohumeral muscular dystrophy (FSHD) is the third most common dystrophy among all dystrophies. In this paper, we report a novel case of FSHD coinciding with ET.

The male FSHD patient was diagnosed at the age of 17 with difficulty of raising his arms. He had 4q35 D4Z4 repeat contraction. A neurological examination revealed positive facial involvement and scapula alata; right and left shoulder flexion 4+; right forearm flexion 4+; left forearm flexion 3+; right and left hip flexion 4+; remaining muscle strengths 5+. Mild involvement and loss of power were seen in the extensor indicis, peroneal muscles, and abdominal muscles. When the patient was 67 years old, he was admitted to the hematology clinic with facial redness and increased platelet count (1,200,000/mm^3^) without hepatosplenomegaly.

**Figure 1:**
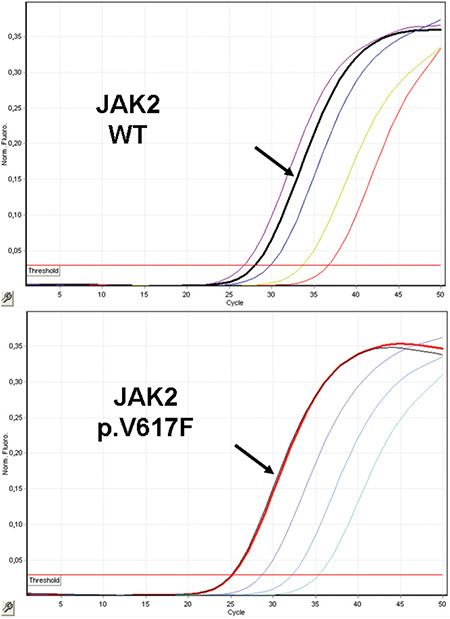
Visual representation of the patient’s quantitative realtime PCR results for the *JAK2* gene.

Since myeloproliferative neoplasms (MPNs) are frequently related to somatic mutations of the *JAK2*, *MPL*, and *CALR* genes, the patient’s blood sample was analyzed for the hot-spot mutations of these genes. The exon 10 region of the *MPL* gene was analyzed for p.W515K/L mutation and the exon 9 region of the *CALR* gene was analyzed for insertion/deletion mutations with PCR/sequencing methods. The exon 14 region of the *JAK2* (Janus kinase 2) gene was investigated for p.V617F (c.1849G>T) mutation by quantitative real-time PCR using plasmids of wild type and mutant alleles. There was no mutation in the target regions of the *MPL* and *CALR* genes. In the exon 14 region of the *JAK2* gene, p.V617F (c.1849G>T) mutation was detected with 28% allele burden (Figure 1).

His child was also investigated for the *JAK2* p.V617F (c.1849G>T) mutation and was found to be negative. Finally, the patient was diagnosed with high-risk ET because of being aged >60 years with *JAK2* mutation, and hydroxyurea and low-dose aspirin were started.

In this case, the presence of the *JAK2* p.V617F mutation confirmed the diagnosis of ET. There has been no report on the co-occurrence of FSHD with MPNs including ET and *JAK2* p.V617F mutation, and this is the first such case in the literature. Recently, a number of reports indicated that some germ-line DNA variants may predispose to MPNs with *JAK2* p.V617F mutation [1].

FSHD patients are prone to develop other systemic diseases, especially malignancies [2], via re-expression of the *DUX4* gene that allows cancer cells to escape immune surveillance [3]. In addition, myeloid cells (including thrombocytes) and skeletal muscle cells originate from the mesoderm. It has been shown that the JAK/STAT pathway is responsible for the proliferation and differentiation of both myeloblasts and myoblast cells [4,5]. Our case might indicate a common mechanism responsible for the development of FSHD and MPNs. In addition to the effect of *DUX4*, since JAK signaling has important functions in the development of both skeletal muscle and thrombocytes, we propose that this co-occurrence signifies the importance of focusing on the role of the JAK/STAT pathway for FSHD pathophysiology, which can also contribute to the molecular mechanisms of ET.
